# Combined with the semantic features of CT and selected clinical variables, a machine learning model for accurately predicting the prognosis of Omicron was established

**DOI:** 10.1093/bjro/tzae013

**Published:** 2024-06-05

**Authors:** Di Jin, Zicong Li, Zhikang Deng, Jiayu Nan, Pei Huang, Bingliang Zeng, Bing Fan

**Affiliations:** Medical Department, Medical College of Nanchang University, Nanchang University, Nanchang 330006, China; Department of Radiology, Jiangxi Provincial People’s Hospital, The First Affiliated Hospital of Nanchang Medical College, Nanchang 330000, China; Department of Radiology, Jiangxi Provincial People’s Hospital, The First Affiliated Hospital of Nanchang Medical College, Nanchang 330000, China; Medical Department, Medical College of Nanchang University, Nanchang University, Nanchang 330006, China; Department of Nuclear Medicine, Jiangxi Provincial People’s Hospital, The First Affiliated Hospital of Nanchang Medical College, Nanchang 330000, China; Medical Department, Medical College of Nanchang University, Nanchang University, Nanchang 330006, China; Department of Radiology, Jiangxi Cancer Hospital, The Second Affiliated Hospital of Nanchang Medical College, Nanchang 330000, China; Medical Department, Medical College of Nanchang University, Nanchang University, Nanchang 330006, China; Department of Radiology, Jiangxi Provincial People’s Hospital, The First Affiliated Hospital of Nanchang Medical College, Nanchang 330000, China; Department of Radiology, Jiangxi Provincial People’s Hospital, The First Affiliated Hospital of Nanchang Medical College, Nanchang 330000, China; Department of Radiology, Jiangxi Provincial People’s Hospital, The First Affiliated Hospital of Nanchang Medical College, Nanchang 330000, China

**Keywords:** omicron, radiomics, non-enhanced computed tomography

## Abstract

**Objectives:**

To efficiently use medical resources and offer optimal personalized treatment for individuals with Omicron infection, it is vital to predict the disease’s outcome early on. This research developed three machine learning models to foresee the results for Omicron-infected patients.

**Methods:**

Data from 253 Omicron-infected patients, including their CT scans, clinical details, and relevant laboratory values, were studied. The patients were categorized into two groups based on their disease progression: favourable prognosis and unfavourable prognosis. Patients manifesting respiratory failure, acute liver or kidney impairment, or fatalities were placed in the “poor” group. Those lacking such symptoms were allocated to the “good” group. The participants were randomly split into training set (202) and validation set (51) with an 8:2 ratio. Radiomics features were produced using image processing, focused segmentation, feature extraction, and selection, leading to the establishment of a radiomics model. A univariate logistic regression method identified potential clinical factors contributing to a clinical model’s development. Eventually, the fused feature set, integrating radiomics features and clinical indicators, was used for the combined model. The model’s prediction performance was assessed using the area under the receiver operating characteristic curve (AUC). The model’s clinical usefulness was evaluated by generating calibration and decision curves.

**Results:**

Compared to other classification models, the combined model showcased the best classification performance. It achieved an AUC of 0.848 and accuracy of 0.763 in the training set, and 0.797 and 0.750 in the validation set, respectively.

**Conclusions:**

This study employed machine learning model to accurately predict the prognosis of Omicron-infected patients.

**Advances in knowledge:**

(1) Topic innovation: At present, there is a lack of research on the use of CT images to construct machine learning models to predict the prognosis of patients with Omicron infection. This study intends to establish clinical, radiomics, and combined models to provide more possibilities for the identification of the two. (2) Platform innovation: The feature extraction and screening and the establishment of omics model in this study will be completed in the intelligent scientific research platform, which can reduce the error caused by human error, simplify the operation steps, and save the time of data processing time.

## Introduction

Omicron, a mutation of SARS-CoV-2, emerged in November 2021. In comparison to other mutated strains, this variant displayed significantly increased transmissibility and immune evasion, rapidly displacing the Delta mutant to become the predominant global epidemic strain in early 2022.^1^^,^^2^ Since July 2022, the globally prevalent Omicron mutants are mainly BA.5 subvariants.^3^ However, with the continuous spread and development of the virus, the transmission advantages of subbranches such as BF.7 and BQ.1, with stronger immune escape and transmission ability, and recombinant mutants (XBB), have rapidly increased. As a result, BA.5.2 has been replaced as the dominant epidemic strain in some countries and regions.^4^ Studies have indicated that Omicron might evade vaccine or prior infection immunity more extensively than any other variant, leading to reduced effectiveness of existing vaccines against Omicron, although immune enhancers could boost immunity.^5^ Early precautionary measures, including vaccination, remain key in curbing Omicron.^5^

Both domestic and international evidence indicates that the lung pathogenicity of Omicron mutants is notably weaker compared to other strains, resulting in most infected patients experiencing mild symptoms.^6^^,^^7^ However, the median latency period may be shorter than that of the original virus.^8^ The clinical presentation has evolved from pneumonia to upper respiratory tract infections, such as runny nose, fatigue, sneezing, sore throat, and so on.^9^^,^^10^ Nonetheless, some patients may progress to severe or even life-threatening diseases.^11^ Therefore, achieving an early predictive diagnosis of patients can assist clinicians in providing personalized treatment, thereby improving prognosis and reducing mortality.

The real-time reverse transcriptase polymerase chain reaction (RT-PCR) of upper and lower respiratory tract samples, such as nasopharyngeal or oropharyngeal swabs, sputum, tracheal aspiration, and bronchoalveolar lavage fluid (BALF), serves as a reference standard for diagnosing Omicron infections.^12^ However, it cannot depict the lung lesions of the patient, and the results may be influenced by the patient’s viral load, sampling method, sampling time, or laboratory issues.^13^^,^^14^ Laboratory tests, such as IgM and IgG, can to some extent reflect the severity of patients’ symptoms.^15^ Nevertheless, there is insufficient clear data to prove the effectiveness of these tests in reflecting disease prognosis. Chest CT is a rapid, non-invasive, and sensitive method widely used in diagnosing COVID-19.^16^^,^^17^ It is also one of the diagnostic criteria for Omicron infection. However, conventional CT images still have limitations when evaluating disease progression and prognosis.

Radiomics involves extracting a substantial number of image features, which are then transformed into data. This method manages to capture both the broader attributes of a disease and the finer microscopic characteristics that healthcare professionals find challenging to discern visually. Combining imaging data with clinical information offers a more comprehensive understanding of a patient’s condition. Clinical data encompass patient history, symptoms, physical exams, and lab tests, aiding in contextualizing imaging findings. Together, this amalgamation can yield a more precise diagnosis and assist clinicians in determining the most suitable treatment pathway.^18^ In essence, this technique holds significant potential for disease classification, identification, and prognosis. Recent work by Wu et al. underscores that models founded on radiomics data furnish crucial insights for COVID-19 prognosis.^19^ Wang et al.’s findings highlight the predictive prowess of their clinical and radiology factor-based model in foreseeing COVID-19 onset.^20^

The aim of this study is to construct a predictive model by amalgamating radiological features and clinical indices extracted from initial CT scans. This model is designed to evaluate the prognosis of individuals with Omicron infection. Beyond enhancing our understanding of the disease, this approach bears substantial clinical implications, ranging from refining patient management to judicious allocation of medical resources and selection of appropriate therapies.

## Method

### Ethics

The data used for this investigation were collected from 253 individuals diagnosed with Omicron infection and hospitalized at Jiangxi Provincial People’s Hospital from December 2022 to January 2023. The study protocol received approval from the review board of the First Affiliated Hospital of Nanchang Medical College. Given the retrospective nature of this research, patient’s informed consent was not required. The study adhered to the principles outlined in the Helsinki Declaration.

### Patient

We accessed the electronic medical record system to compile case information, encompassing demographic data, primary illnesses, laboratory examinations, and CT images from the picture archiving and communication system (PACS). For statistical analysis and modelling, we selected initial laboratory assessments and CT image data collected during patients’ hospital stays. Study eligibility criteria comprised: (a) confirmed COVID-19 diagnosis via RT-PCR, (b) accessible chest CT imaging data, and (c) available baseline details. Exclusion criteria encompassed: (a) significant motion artifacts in CT images, (b) minor or indistinct lesions undetectable by CT, (c) absence of imaging or clinical data, or(d) previous lung illness. A total of 45 patients were excluded. The enrolled subjects were categorized into poor-prognosis and good-prognosis groups based on disease progression. Patients manifesting respiratory failure, acute liver or kidney impairment, or fatalities were placed in the “poor” group. Those lacking such symptoms were allocated to the “good” group. This classification method mirrors approaches employed in previous studies for assessing pneumonia severity.^21^^,^^22^

### Scanning equipment

Chest CT scans were conducted under breath-holding conditions to diminish motion artifacts. Images were captured using GE Optima 660, Philips ICT, and GE Revolution scanners. Patients assumed a supine posture with their heads inclined forward, undergoing continuous scans from the top to the bottom of their lungs. During CT imaging, the tube voltage was set at 120kVp, and the tube current was 210 mA. A standard algorithm was utilized for thin-layer reconstruction, yielding a 1.25 mm thickness. Intravenous contrast media was not administered to any patients.

### Image processing and focus segmentation

For characteristic viral pneumonia feature extraction, two radiologists with over five years of experience in chest imaging diagnosis utilized the Darwin platform to identify lesions and manually delineate the region of interest (ROI). Suspicious lesions were confirmed by a third senior doctor. Lesions smaller than 3 mm in diameter, along with other bacterial infections, pulmonary tuberculosis, and suspected neoplastic lesions were excluded. During this process, all doctors were unaware of the patient groupings.

### Feature extraction, selection, and model establishment

**Figure 1.**
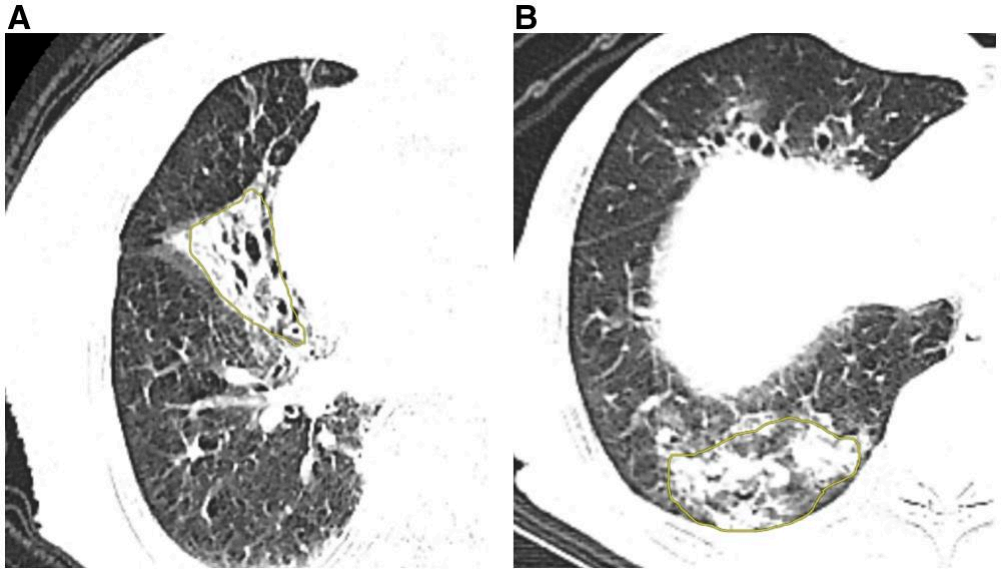
Regions of interest (ROIs) for both the “poor” groups (A) and the “good” groups (B). These ROIs were manually delineated on the NE-CT scans utilizing an image processing software (DARWIN intelligent scientific research platform). The outlines were then amalgamated to form a diagram of the region of interest (ROI), highlighted in yellow.

**Figure 2.**
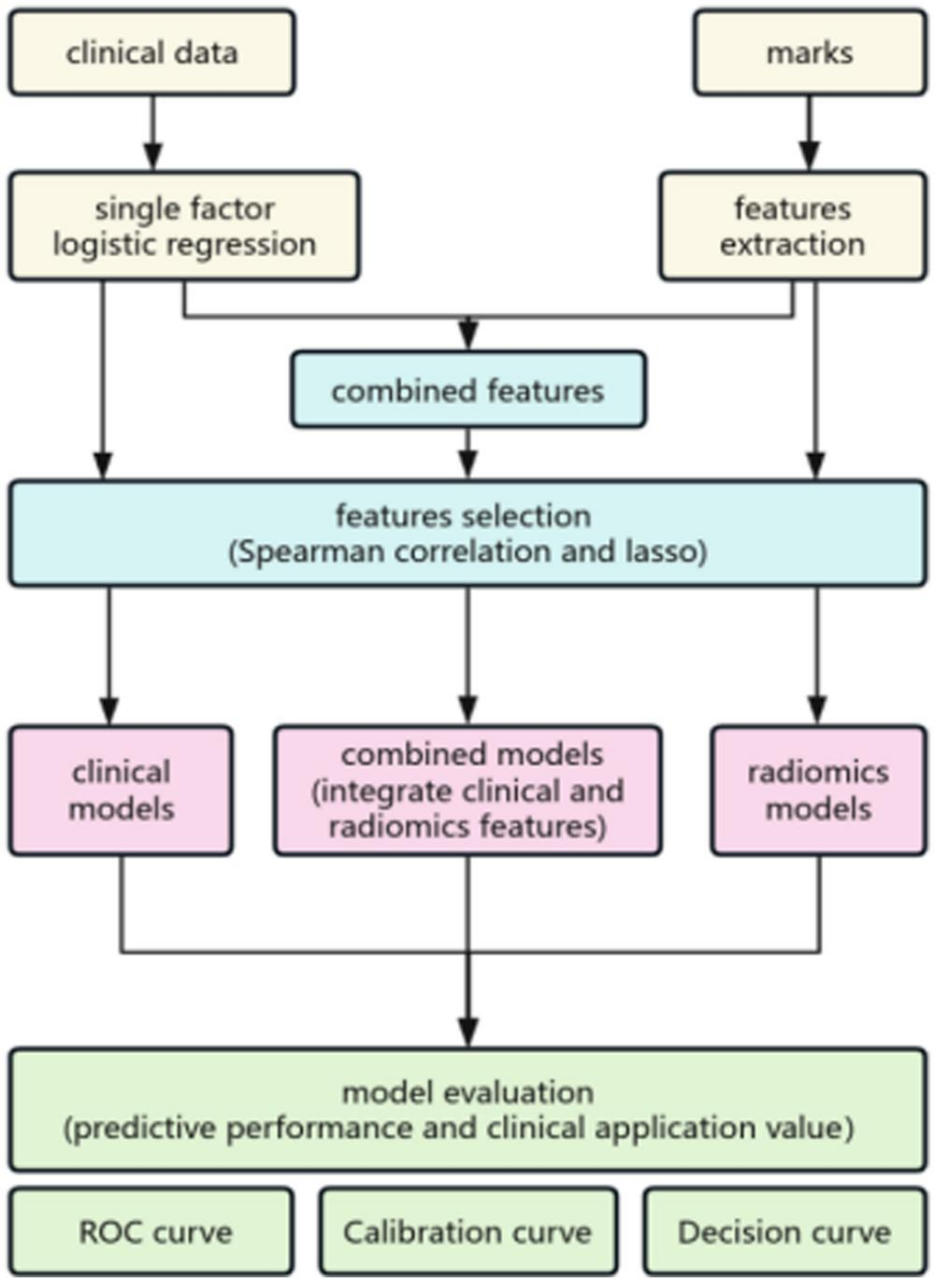
Workflow. Abbreviation: ROC = receiver operating characteristic.

The Darwinian intelligent scientific research platform was used to extract combinatorial features from the marked ROI images, followed by preprocessing to develop a suitable model (Figures 1 and 2). The optimal feature filter assessed the linear correlation between each feature and the category label, leading to the selection of relevant features. Simultaneously, univariate analysis gauged the relationship among clinical factors, underlying diseases, laboratory indices, and prognosis, aiding in identifying relevant factors.

Using concatenation, radiomics features and clinical features were merged into a compound feature vector, further culminating in a fusion feature set. Following an 8:2 ratio, the fused feature set was divided into training and testing subsets. The filtered dataset were randomly allocated, with 80% assigned to the training group (146 good prognosis and 56 poor prognosis cases) and 20% to the verification group (39 good prognosis and 12 poor prognosis cases). The first step involved standardizing and dimensionality reduction of features. Features with high correlation (corr > 0.9) were evaluated based on their correlation coefficient, with one being retained among highly correlated features.

**Figure 3.**
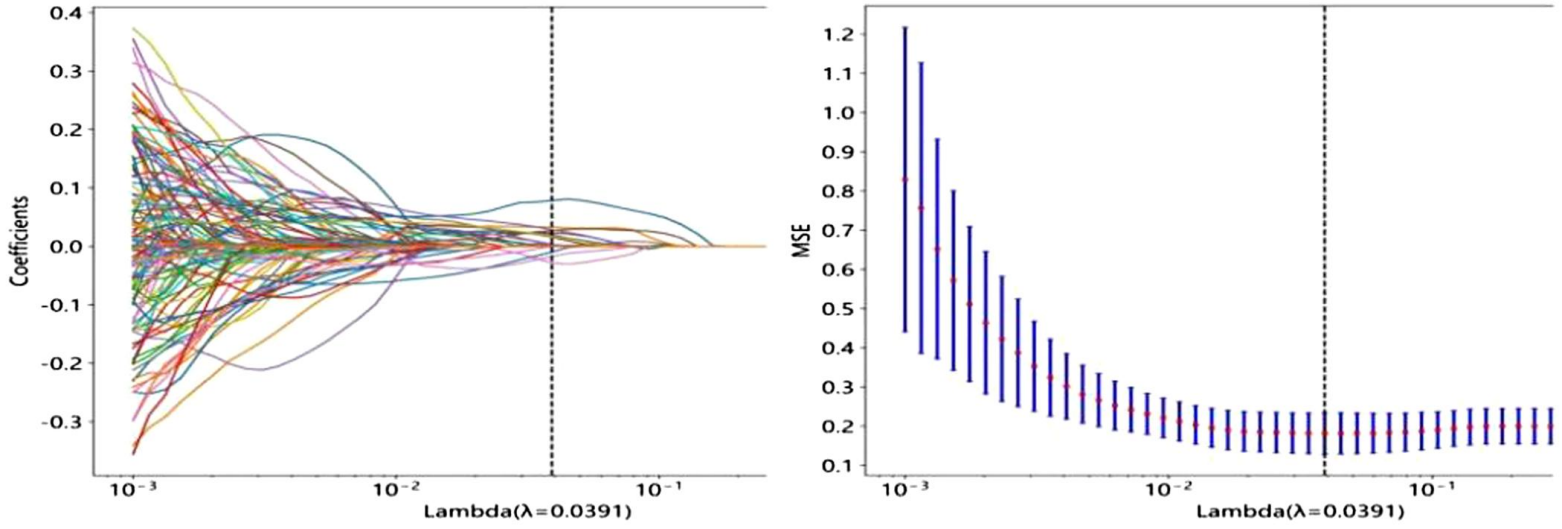
Utilizing the LASSO algorithm for feature selection (left: LASSO path; right: MSE path).

**Figure 4.**
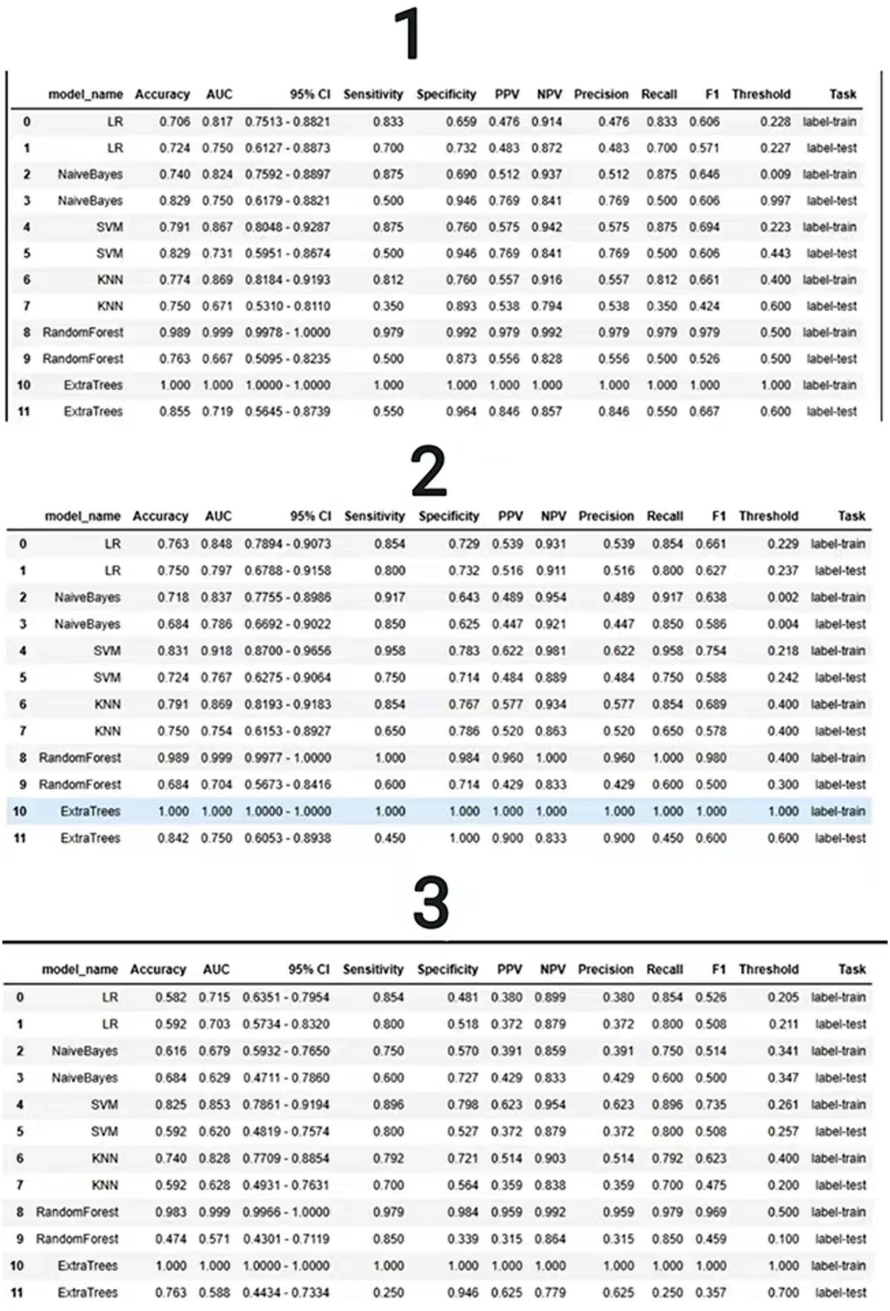
Accuracy and AUC of the different models (SVM, RF, ET, NB, RF, KNN): 1: radiomic model, 2: fusion model, 3: clinical model. Abbreviations: KNN = k-Nearest Neighbour; LR = linear regression; SVM = Support Vector Machine.

For feature selection, we utilized logistic regression with Lasso regularization. The Lasso regularization technique imposes a penalty on the absolute size of the regression coefficients, promoting sparsity in the resulting model. Specifically, we applied Lasso logistic regression to the dataset to identify the most significant features by minimizing the negative log-likelihood loss function with an added L1 penalty term on the regression coefficients. By tuning the regularization parameter through cross-validation, we obtained optimal feature selection, retaining only those features with non-zero coefficients. This approach guarantees reproducibility and transparency in the feature selection process, as the regularization parameter value and feature selection criteria can be explicitly specified (Figure 3). Subsequently, six classifiers, including Support Vector Machine (SVM), Random Forest (RF), Extra Trees (ET), Naive Bayes (NB), another RF, and k-Nearest Neighbour (KNN), were developed, leading to the establishment of the training set model (Figure 4).

The prediction model’s effectiveness was assessed using the area under the receiver operating characteristic curve (AUC), where a larger AUC signifies higher prediction accuracy. Calibration curves were employed to evaluate the association between predicted risks and actual outcomes. Additionally, the model’s clinical utility was determined via decision curve analysis (DCA).^20^

### Statistical analysis

Univariate logistic regression analysis was used to examine independent predictors of favourable or unfavourable Omicron infection prognosis, encompassing clinical manifestations, laboratory parameters, and more. Statistical significance was considered when *P* < .05. AUC calculation and correction curve establishment were performed, and the model’s clinical application value was evaluated through DCA. All statistical analyses and visualizations were conducted using Python (version 3.9.7).

## Result

A significant difference (*P* < .05) was observed between the “poor” and “good” groups within the training cohort.

### Clinical characteristics

The study incorporated data from a total of 253 patients, wherein 202 patients constituted the training set and the remaining 51 patients comprised the validation set. Patient characteristics for both sets are detailed in Table 1. The average age of the patients was 68 years. Of the 253 patients, 160 were male and 93 were female. Notable differences in gender, albumin (paired: 0.116), and lymphocyte count (paired: 0.119) between the training and validation groups were absent (Table 1). Within the training group, notable distinctions in D-dimer levels, age, diabetes presence, history of surgery, cerebrovascular disease occurrence, as well as occurrences of chest tightness and shortness of breath were observed between individuals with good prognosis and poor prognosis (*P* < .05).

**Table 1.** Characteristics of the training and validation cohorts are presented.

Feature name	Label all	Label 0	Label 1	*P* value
Age	68.53 + 17.13	66.46 + 17.76	74.13 + 13.92	0.001
Gender				0.240
Male	160 (63.24)	113 (61.08)	47 (69.12)	
Female	93 (36.76)	72 (38.92)	21 (30.88)	
Albumin	34.17 + 9.79	34.76 + 10.84	32.57 + 5.88	0.116
D-dimer	1.10 + 1.99	0.97 + 1.80	1.45 + 2.43	0.09
Lymphocyte	17.99 + 16.33	18.96 + 18.08	15.35 + 9.74	0.119
Surgical				0.012
0	164 (64.82)	111 (60.00)	53 (77.94)	
1	89 (35.18)	74 (40.00)	1522.06)	
Cerebrovascular				0.08
0	209 (82.61)	158 (85.41)	51 (75.00)	
1	44 (17.39)	27 (14.59)	17 (25.00)	
Anhelation				0.005
0	150 (59.29)	120 (64.86)	30 (44.12)	
1	103 (40.71)	65 (35.14)	38 (55.88)	
Diabetes				0.003
1	68 (26.88)	40 (21.62)	28 (41.18)	

### Combinatorial features

A total of 1032 associated features, comprising first-order, texture, and shape-based features, were extracted using the Darwin intelligent scientific research platform. These features encompassed 9 shape-related, 18 first-order, 24 GLCM features, 14 GLDM features, 16 GLRLM features, 16 GLSZM features, and 5 NGTDM features. After meticulous screening, nine pertinent features were retained.

**Figure 5.**
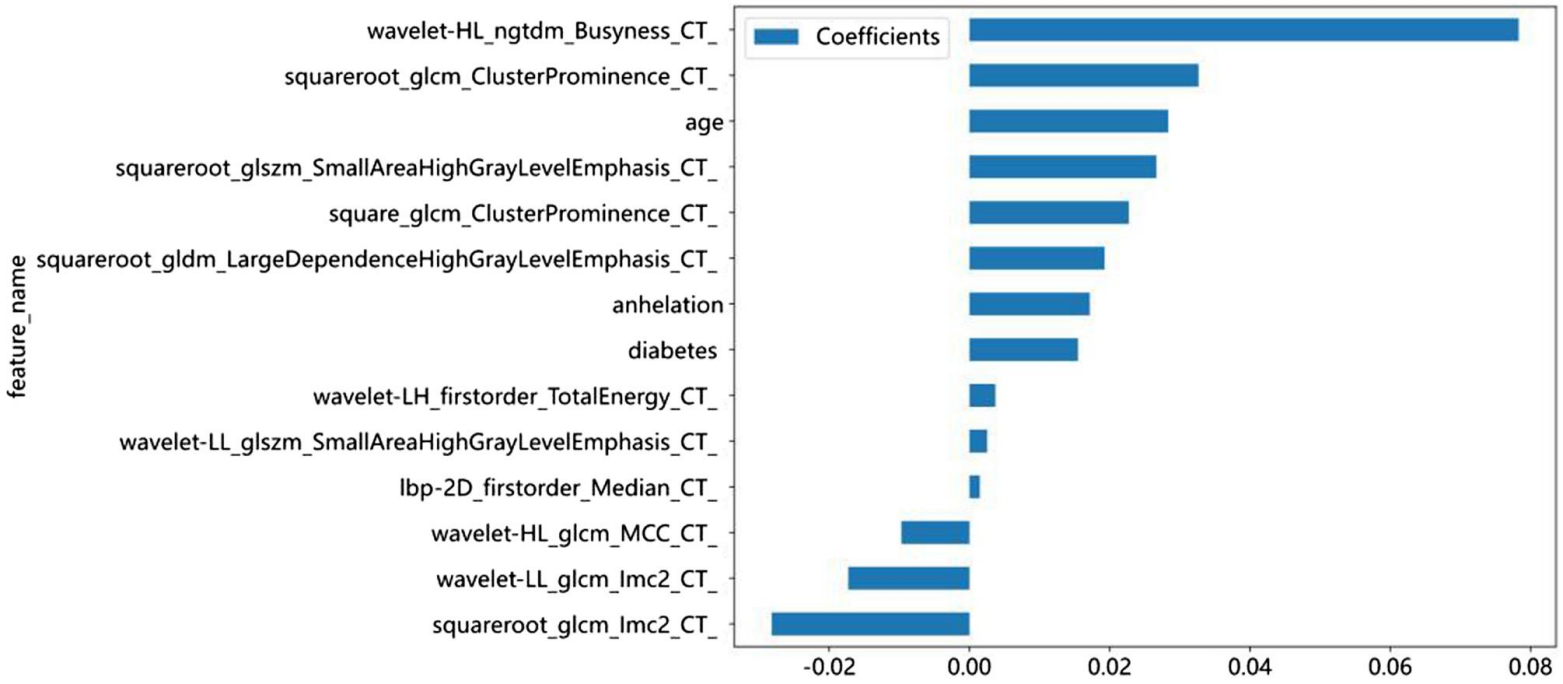
The final 14 features selected (11 taxonomic features and 3 clinical features).

For fusion features, 170 features underwent screening via the Spearman correlation coefficient, followed by lasso regression. Within the training set, 14 features were chosen based on the optimal L1-regularization parameter and corresponding coefficients, including 11 taxonomic features and 3 clinical features (Figure 5).

### Model evaluation

**Figure 6.**
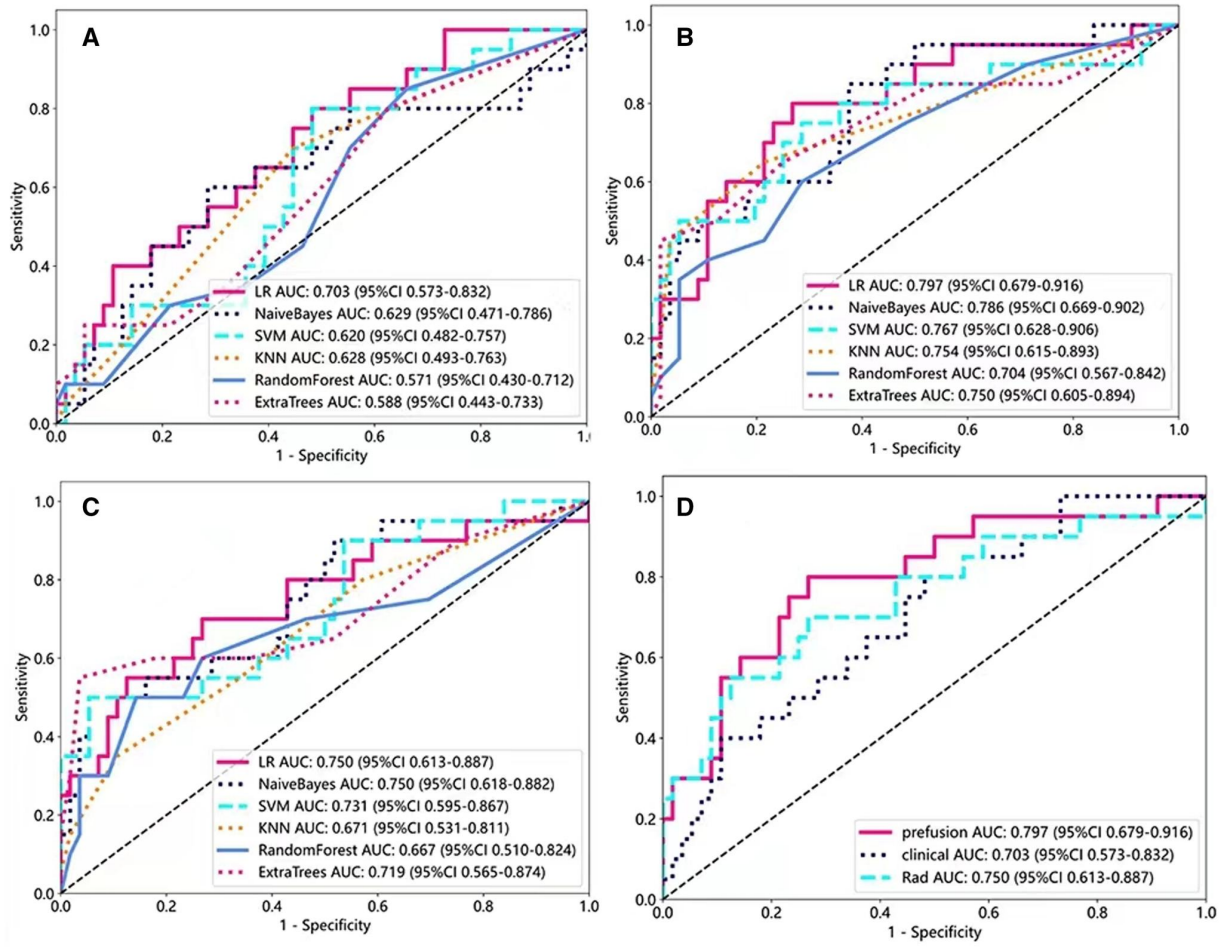
ROC curve of the different models: (A): clinical model, (B): radiomic model, (C): fusion model, (D): three model contrasts. Abbreviations: AUC = area under the receiver operating characteristic curve; KNN = k-Nearest Neighbour; LR = linear regression; SVM = Support Vector Machine.

In the clinical model, logistic regression achieved the highest AUC, reaching 0.715 in the training dataset and 0.703 in the test dataset. Within the group model, Naive Bayes exhibited a superior AUC compared to other models, attaining values of 0.824 in the training dataset and 0.750 in the test dataset. In the combination model, logistic regression outperformed alternative classifiers, yielding AUC values of 0.848 in the training dataset and 0.797 in the test dataset. Among these three models, the combined model’s logistic regression yielded the most favourable AUC value, accompanied by a test dataset accuracy of 0.750 (refer to Figure 6).

**Figure 7.**
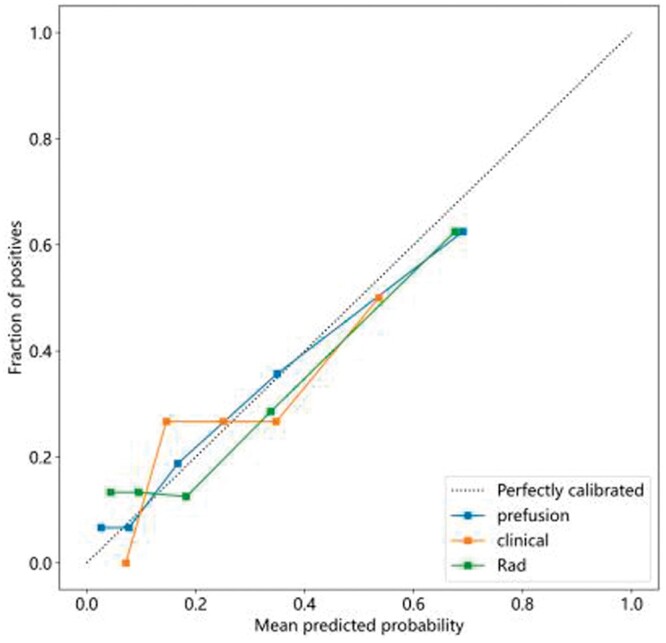
Evaluation of the calibration curves for clinical, radiomics, and fusion models.

**Figure 8.**
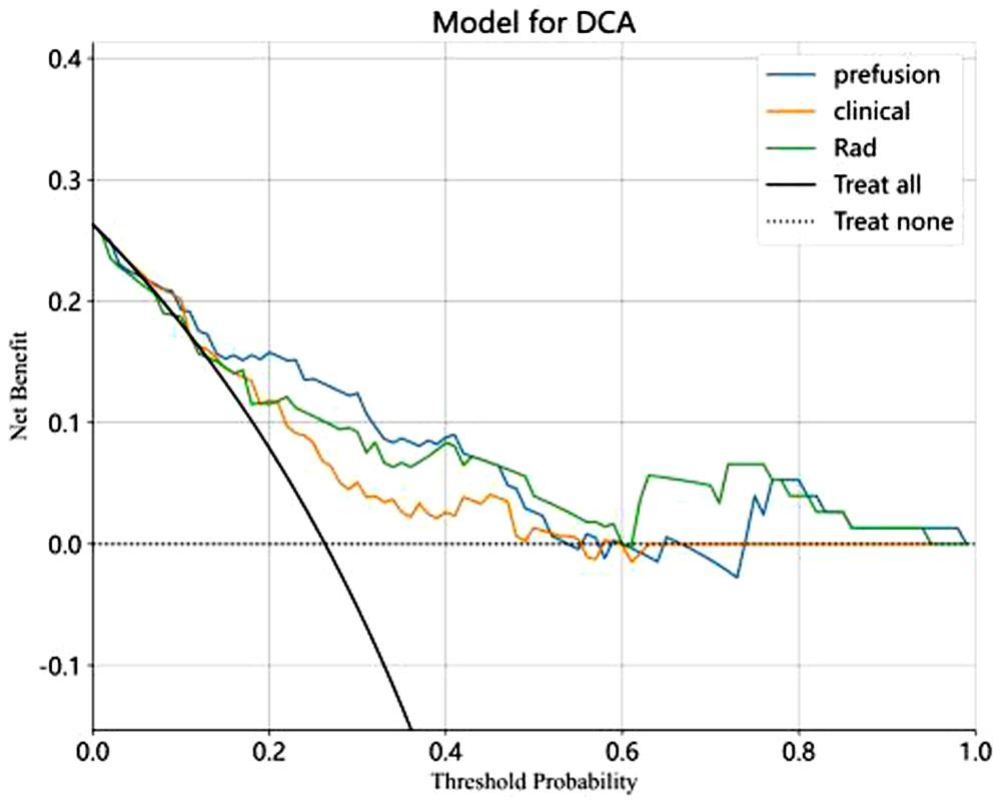
Clinical, radiomics, and fusion models’ decision curve assessment (DCA).

The calibration curve showcases a robust alignment between the predicted value of the integrated model and the optimal value, as indicated in Figure 7. The same figure further illustrates the calibration curves for the three models. To appraise the model’s performance, a DCA is executed. Notably, when the threshold probability dips below 40%, the fusion model offers more substantial advantages compared to other models, signifying a positive clinical influence (refer to Figure 8).

## Discussion

In our study, we identified 11 radiological attributes and 3 clinical factors that exhibited notable associations with the prognosis of novel coronavirus infection. Leveraging radiological features and clinical indicators extracted from initial CT images, we devised and validated three machine learning models for disease prognosis. Our findings underscore the efficacy of the integrated model in proficiently forecasting outcomes for Omicron infection patients, surpassing the performance of both fundamental radiological and clinical models.

Omicron variants have the potential to inflict harm on various organs, with severe cases even culminating in mortality. Hence, precise and timely prognosis prediction aids clinicians in crafting tailored treatment strategies, preventing malignant progression in a timely manner, and averting unnecessary resource depletion. Zhou et al.’s research^23^ hints at the capacity of relevant laboratory indices to predict Omicron infection patient prognosis to some extent. However, their scope is confined to specific clinical data without incorporating fused image features. Jayachandran et al. explored the utility of routine chest CT severity in assessing short-term COVID-19 prognosis,^24^ while Shi et al. developed an imaging roadmap for severe pneumonia identification.^25^ These investigations suggest the applicability of imaging for evaluating pneumonia severity and prognosis.

Early intelligent diagnosis has traditionally leaned on artificially designed feature templates or single traditional machine learning techniques.^26^ Intelligent diagnosis is essentially a classification challenge. However, the single-feature machine learning approach, though adept at recognizing specific image feature changes, lacks sensitivity to alterations in other features. When disparities in certain features between two images are minimal, classifiers trained on single features struggle to furnish precise classification results.^27^ Additionally, image quality substantially influences machine recognition and classification, elevating the complexity of classifier training while diminishing classification accuracy.^28^ Conversely, our proposed method harnesses fused features to complement one another, effectively surmounting the limitations intrinsic to single features.

In this study, we utilized the LASSO algorithm to select 11 radiological characteristics, with NGTDM demonstrating the highest predictive accuracy for Omicron infection prognosis. Compared to other image features, NGTDM features effectively capture internal organizational structure changes. They describe the grayscale differences between pixels in a digital image, indicating alterations in internal region structures.

NGTDM attributes encompass roughness, busyness, intensity, contrast, and complexity. Multiple studies have validated their precision. Gökçen et al.^29^ demonstrated NGTDM’s capacity for representing spatial variation in pixel intensity. Liu et al.^30^ indicated that higher intensity NGTDM values imply gradual image intensity changes, yet substantial gray intensity roughness. Although the model combines radiomic features, limited information exists about their function in the model and potential biological foundations, necessitating further research to unveil their potential significance.

Our investigation suggests radiology’s value in assessing Omicron infection prognosis. Based on initial CT scans post-admission, we accurately predicted patient outcomes and intervened as needed. Fusion characteristics were employed in our research to forecast Omicron infection prognosis. This model, based on clinical independent factors and histological traits, is non-invasive, convenient, and rapid. CT radiology image analysis objectively evaluates lesion and organ heterogeneity, offering more precise information on tissue microenvironments than subjective visual assessments.

Nonetheless, our study has limitations. It represents a single centre and may not encompass the entire patient population. The retrospective allocation of patient cohorts from those with hospitalization indicators could introduce selection bias. Our research precision requires improvement. Thus, forthcoming studies will seek to merge multicentre data to bolster findings and gather a larger number of cases for enhanced investigation accuracy.^31^

In conclusion, our study proposes a multi-feature fusion-based machine learning model approach for predicting Omicron infection outcomes. We identified radiological characteristics and clinical indicators significantly associated with novel coronavirus infection prognosis. In the final machine learning model, logistic regression emerged as the best predictor, accurately forecasting Omicron infection prognosis from CT plain scan images.

## Funding 

This work is supported by the National Natural Science Foundation of China (Grants No. 82160335).

## Conflicts of interest 

None declared.

## References

[1] Pascarella S , Ciccozzi M, Bianchi M, Benvenuto D, Cauda R, Cassone A. The electrostatic potential of the Omicron variant spike is higher than in Delta and Delta-plus variants: a hint to higher transmissibility. J Med Virol. 2022;94(4):1277–1280. 34914120 10.1002/jmv.27528

[2] Singhal T. The emergence of Omicron: challenging times are here again. Indian J Pediatr. 2022;89(5):490–496. 35025038 10.1007/s12098-022-04077-4 PMC8756165

[3] Rana R , Kant R, Huirem RS, Bohra D, Ganguly NK. Omicron variant: current insights and future directions. Microbiol Res. 2022;265:127204. 36152612 10.1016/j.micres.2022.127204 PMC9482093

[4] Cocherie T , Zafilaza K, Leducq V, et al Epidemiology and characteristics of SARS-CoV-2 variants of concern: the impacts of the spike mutations. Microorganisms. 2023;11(1):30. 10.3390/microorganisms11010030 PMC9866527 36677322

[5] Callaway E. Omicron likely to weaken COVID vaccine protection. Nature. 2021;600(7889):367–368. 34880488 10.1038/d41586-021-03672-3

[6] Kupferschmidt K , Vogel G. How bad is Omicron? Some clues are emerging. Science. 2021;374(6573):1304–1305. 34882443 10.1126/science.acx9782

[7] Guo Y , Han J, Zhang Y, et al SARS-CoV-2 Omicron variant: epidemiological features, biological characteristics, and clinical significance. Front Immunol. 2022;13:877101. 35572518 10.3389/fimmu.2022.877101 PMC9099228

[8] Jansen L , Tegomoh B, Lange K, et al Investigation of a SARS-CoV-2 B.1.1.529 (Omicron) variant cluster—Nebraska. MMWR Morb Mortal Wkly Rep. 2021;70(5152):1782–1784. 34968376 10.15585/mmwr.mm705152e3 PMC8736273

[9] Iacobucci G. Covid-19: runny nose, headache, and fatigue are commonest symptoms of omicron, early data show. BMJ. 2021;375:n3103. 34916215 10.1136/bmj.n3103

[10] Meo SA , Meo AS, Al-Jassir FF, Klonoff DC. Omicron SARS-CoV-2 new variant: global prevalence and biological and clinical characteristics. Eur Rev Med Pharmacol Sci. 2021;25(24):8012–8018. 34982465 10.26355/eurrev_202112_27652

[11] Nealon J , Cowling BJ. Omicron severity: milder but not mild. Lancet. 2022;399(10323):412–413. 35065007 10.1016/S0140-6736(22)00056-3 PMC8769661

[12] Bazargan M , Elahi R, Esmaeilzadeh A. OMICRON: virology, immunopathogenesis, and laboratory diagnosis. J Gene Med. 2022;24(7):e3435. 35726542 10.1002/jgm.3435 PMC9350010

[13] Mohseni Afshar Z , Tavakoli Pirzaman A, Karim B, et al SARS-CoV-2 Omicron (B.1.1.529) variant: a challenge with COVID-19. Diagnostics (Basel). 2023;13(3):559. 36766664 10.3390/diagnostics13030559 PMC9913917

[14] Zou L , Ruan F, Huang M, et al SARS-CoV-2 viral load in upper respiratory specimens of infected patients. N Engl J Med. 2020;382(12):1177–1179. 32074444 10.1056/NEJMc2001737 PMC7121626

[15] Espejo AP , Akgun Y, Al Mana AF, et al Review of current advances in serologic testing for COVID-19. Am J Clin Pathol. 2020;154(3):293–304. 32583852 10.1093/ajcp/aqaa112 PMC7337672

[16] Ai T , Yang Z, Hou H, et al Correlation of chest CT and RT-PCR testing for coronavirus disease 2019 (COVID-19) in China: a report of 1014 cases. Radiology. 2020;296(2):E32–E40. 32101510 10.1148/radiol.2020200642 PMC7233399

[17] Revel MP , Parkar AP, Prosch H, et al European Society of Radiology (ESR) and the European Society of Thoracic Imaging (ESTI). COVID-19 patients and the radiology department—advice from the European Society of Radiology (ESR) and the European Society of Thoracic Imaging (ESTI). Eur Radiol. 2020;30(9):4903–4909. 32314058 10.1007/s00330-020-06865-y PMC7170031

[18] Lubner MG , Smith AD, Sandrasegaran K, Sahani DV, Pickhardt PJ. CT texture analysis: definitions, applications, biologic correlates, and challenges. Radiographics. 2017;37(5):1483–1503. 28898189 10.1148/rg.2017170056

[19] Wu S , Zhang R, Wan X, et al Chest computed tomography radiomics to predict the outcome for patients with COVID-19 at an early stage. Diagn Interv Radiol. 2023;29(1):91–102. 36960545 10.5152/dir.2022.21576 PMC10679604

[20] Wang D , Huang C, Bao S, et al Study on the prognosis predictive model of COVID-19 patients based on CT radiomics. Sci Rep. 2021;11(1):11591. 34078950 10.1038/s41598-021-90991-0 PMC8172890

[21] Gao HN , Lu HZ, Cao B, et al Clinical findings in 111 cases of influenza A (H7N9) virus infection. N Engl J Med. 2013;368(24):2277–2285. 23697469 10.1056/NEJMoa1305584

[22] Zhang M , Zeng X, Huang C, et al An AI-based radiomics nomogram for disease prognosis in patients with COVID-19 pneumonia using initial CT images and clinical indicators. Int J Med Inform. 2021;154:104545. 34464848 10.1016/j.ijmedinf.2021.104545 PMC8353975

[23] Zhou Y , Zhang M, Wu X, et al Platelet-albumin-bilirubin score and neutrophil-to-lymphocyte ratio predict intensive care unit admission in patients with end-stage kidney disease infected with the Omicron variant of COVID-19: a single-center prospective cohort study. Ren Fail. 2023;45(1):2199097. 37051667 10.1080/0886022X.2023.2199097 PMC10114985

[24] Jayachandran AK , Nelson V, Shajahan ME. Chest CT severity score as a predictor of mortality and short-term prognosis in COVID-19. J Family Med Prim Care. 2022;11(8):4363–4367. 36353028 10.4103/jfmpc.jfmpc_209_22 PMC9638539

[25] Shi H , Xu Z, Cheng G, et al CT-based radiomic nomogram for predicting the severity of patients with COVID-19. Eur J Med Res. 2022;27(1):13. 35078525 10.1186/s40001-022-00634-x PMC8787184

[26] Turchin A , Shubina M, Breydo E, Pendergrass ML, Einbinder JS. Comparison of information content of structured and narrative text data sources on the example of medication intensification. J Am Med Inform Assoc. 2009;16(3):362–370. 19261947 10.1197/jamia.M2777 PMC2732236

[27] Srinivasan PP , Kim LA, Mettu PS, et al Fully automated detection of diabetic macular edema and dry age-related macular degeneration from optical coherence tomography images. Biomed Opt Express. 2014;5(10):3568–3577. 25360373 10.1364/BOE.5.003568 PMC4206325

[28] Alsaih K , Lemaitre G, Rastgoo M, Massich J, Sidibé D, Meriaudeau F. Machine learning techniques for diabetic macular edema (DME) classification on SD-OCT images. Biomed Eng Online. 2017;16(1):68. 28592309 10.1186/s12938-017-0352-9 PMC5463338

[29] Çetinel G , Mutlu F, Gül S. Decision support system for breast lesions via dynamic contrast enhanced magnetic resonance imaging. Phys Eng Sci Med. 2020;43(3):1029–1048. 32691326 10.1007/s13246-020-00902-2

[30] Liu J , Xu H, Qing H, et al Comparison of radiomic models based on low-dose and standard-dose CT for prediction of adenocarcinomas and benign lesions in solid pulmonary nodules. Front Oncol. 2020;10:634298. 33604303 10.3389/fonc.2020.634298 PMC7884759

[31] Pasini G , Bini F, Russo G, Comelli A, Marinozzi F, Stefano A. matRadiomics: a novel and complete radiomics framework, from image visualization to predictive model. J Imaging. 2022;8(8):221. 36005464 10.3390/jimaging8080221 PMC9410206

